# Antibiotics in Wastewater: Baseline of the Influent and Effluent Streams in Kuwait

**DOI:** 10.3390/toxics10040174

**Published:** 2022-04-01

**Authors:** Bondi Gevao, Saif Uddin, Divya Krishnan, Smitha Rajagopalan, Nazima Habibi

**Affiliations:** 1 Environmental Protection Authority, Freetown 47235, Sierra Leone; bondi.gevao@epa.gov.sl; 2 Environment and Life Sciences Research Center, Kuwait Institute for Scientific Research, Safat 13109, Kuwait; dkrishnan@kisr.edu.kw (D.K.); srajagopalan@kisr.edu.kw (S.R.); nhabibi@kisr.edu.kw (N.H.)

**Keywords:** pharmaceuticals, antibiotics, influent, effluent, removal efficiency, WWTP

## Abstract

This study provides baseline information on the concentrations of antibiotics in influent and effluent from two wastewater treatment plants in regular operation in the State of Kuwait. Wastewater samples were collected from the influent and effluent streams of two WWTPs, over four sampling campaigns and analyzed for a broad range of antibiotics. The mean influent concentrations of sulfamethoxazole, ciprofloxacin, clarithromycin, and cefalexin were 852 ng/L, 672 ng/L, 592 ng/L), and 491 ng/L, respectively, at Umm Al Hayman WWTP. At the Kabd WWTP, the influent concentration of clarithromycin was highest with a mean of 949 ng/L, followed by ciprofloxacin (mean, 865 ng/L), cefalexin (mean, 598 ng/L), and sulfamethoxazole (mean, 520 ng/L). The dominant compounds in the effluent from Umm Al Hayman were sulfamethoxazole (mean, 212 ng/L), ciprofloxacin (mean, 153 ng/L), ofloxacin (mean, 120 ng/L), dimetridazole (mean, 96 ng/L), and metronidazole (mean, 93 ng/L). Whereas, at the Kabd WWTP, the dominant compounds were sulfamethoxazole (mean, 338 ng/L), dimetridazole (mean, 274 ng/L), cefalexin (mean, 213 ng/L), ciprofloxacin (mean, 192 ng/L), and clarithromycin (189 ng/L). The mean influent concentrations of all compounds were higher than those measured in the effluents. The concentrations of antibiotic compounds were not significantly different between the two WWTPs (*p* > 0.05). The removal efficiencies of the various antibiotics over the four sampling campaigns for the Kabd and Umm Hayman WWTPs ranged between 10.87 and 99.75% and also showed that they were variable and were compound dependent. The data clearly show that the concentrations of antibiotics measured in the influents of both WWTPs were highest in samples collected during the winter-summer (September samples) transition followed by the concentrations measured during the winter-summer (March samples) transition period. This is possibly linked to the increased prescription of these medications to treat infectious diseases and flu prevalent in Kuwait during these periods. This study provides the first reported concentrations of antibiotics in the dissolved aqueous influents and effluents of WWTPs in Kuwait. Additional studies are required to evaluate the environmental impact that antibiotic residues may cause since treated wastewater is used in irrigation, and often there are instances when untreated wastewater is discharged directly into the marine environment.

## 1. Introduction

Antibiotics are a group of pharmaceuticals used to cure or prevent infectious human and veterinary diseases. After systemic absorption due to topical, pulmonary, or ingestion, residues of the parent pharmaceutical (and in some cases a complex array of metabolites) are excreted in urine and feces or are dislodged from the skin by sweating and bathing. These excreted compounds eventually end up in wastewater treatment plants and aquatic environments [1,2,3,4,5,6,7,8,9,10,11,12,13,14,15,16,17,18,19]. The primary route by which human-use antibiotics gain entry to the environment is from the effluent from wastewater treatment plants after excretion from the body [20,21,22,23,24,25,26,27,28,29,30,31,32,33]. In addition to the excretion of pharmaceutical residues after metabolism, disposal of expired or unwanted drugs in sinks and toilets move them into sewers and eventually into the wastewater treatment plants [34]. Although wastewater treatment plants are not specifically designed to remove antibiotics and other micropollutants [14,35,36,37,38,39,40,41,42], there is often recorded a significant difference in concentrations between influent and effluent streams. The concentration in the effluent usually enters the aquatic environment, where concerns emanate about the potential for the facilitation or development of antibiotic-resistant bacteria and antibiotic-resistant genes [43,44,45,46,47,48,49,50,51,52]. It has been suggested in several previous studies that the development of antibiotic resistance by bacteria was due to the occurrence of subtherapeutic concentrations of these antibiotics in these environments [53,54]. Several studies have reported measurable concentrations of antibiotics in the WWTP effluents that eventually ended up in surface waters [55,56,57,58] or even in the food chain [59] following recent and frequent applications of treated wastewater in aquaculture and agriculture [60]. There are many studies on antibiotics released from WWTPs in Europe [61,62], North America, the East, and the Far East [63,64], but very few assessments have been carried out in the Middle East and Africa [11,65,66,67]. This is the first study to examine the occurrence and concentrations of a broad range of antibiotics in the influent and effluent wastewater streams at two major WWTPs in Kuwait under normal operations. The WWTPs in this study both operate conventional activated sludge biological treatments with slightly different principles and both treatment processes disinfect final effluents by chlorination followed by UV irradiation as a tertiary step in the treatment process before effluents are sent to a gathering center for use in agriculture or greenery and landscaping activities [40]. Table 1 details the operating conditions of the two WWTPs in this study.

## 2. Materials and Methods

### 2.1. Sample Collection

The influent samples were taken before the screen when the wastewater entered the plant and the effluent wastewater samples were collected after the disinfection, from two WWTPs in normal operation in Kuwait: Kabd (29°12′18″ N, 047°43′8″ E) in the north and Umm Hayman WWTP in the south (28°52′24″ N, 048°13′57″ E). Primary treatment at Umm Al Hayman involves the removal of floating materials by an impinger employing three-bar screens and grit chambers to remove fine materials. At Kabd, a third step in the primary process involves oil skimmer channels to remove oil and grease before subjecting the effluent to biological treatment. Biological treatment at the Kabd treatment plant is achieved through four vertical-loop reactor aeration systems. Each consists of an aerated anoxic tank followed by an aerobic tank in two parallel operating trains. Biological treatment is based on an extended aeration principle served by four oxidation ditches and final settlement tanks at the Umm AL Hayman treatment plant. The water is then filtered through twelve sand filters, whereas at the Kabd WWTP, filtration is achieved by disc filtration. The water at both WWTPs is treated to the tertiary level with disinfection achieved by chlorination and UV treatment.

Samples were collected in September and December 2013, and March and June 2014. The samples were 24-hour time-proportional composite samples collected using automated wastewater samplers (Sigma 900, HACH, Loveland, CO, USA) installed at the influent and the final effluents. These samplers were programed to sample at 20 min intervals. The samples were collected in 1 L manufacturer-certified clean amber glass bottles rinsed with ultrapure water prior to sample collection. The collected samples were stored in ice, transported to the laboratory, and immediately filtered through 0.45 µm nylon membrane filters (Whatman, Little Chalfont, Buckinghamshire, UK), and 1 g sodium azide was added to each bottle as a preservative. An additional 25 mg/L of ascorbic acid was added to the effluent sample as a quenching agent. The samples were stored at 4 °C prior to their shipment to a laboratory in Spain for analysis. Frozen blue ice packs were used to keep the samples cool during shipment.

### 2.2. Analytical Methods

Details of the analytical methods used for the analysis of the samples, including quality control measures, are given in Gros et al. [19]. The filtered samples were spiked with a range of isotopically labeled standards and extracted using Oasis HLB (60 mg, 3 mL) (Waters Corporation, Milford, MA, USA). The 60 mg cartridges were conditioned with 5 mL of methanol followed by 5 mL of high-performance liquid chromatography (HPLC) grade water at a flow rate of 2 mL/min. In contrast, the 200 mg cartridges were conditioned with 6 mL of methanol followed by 6 mL of HPLC grade water at the same flow rate. Extractions of influent (25 mL) and effluent (50 mL) wastewater, as well as potable water and seawater (500 mL) were carried out automatically by a GX-271 ASPEC™ system (Gilson, Villiers le Bel, France). The analytes were eluted with 6 mL of pure methanol at a rate of 1 mL/min following several rinsing and drying steps. The extracts were concentrated under a gentle stream of nitrogen and reconstituted with 1 mL of methanol/water (10:90, *v*/*v*), spiked with isotopically labeled internal standards. The method development is provided as Tables S1 and S2 in Supplementary Material. A Waters Acquity ultra-performance liquid chromatographic (UPLC) system was used for chromatographic separation and mass spectrometric (MS) detection and analysis on a 5500 QTRAP hybrid triple-linear ion trap system connected in tandem. The wastewater samples were also spiked with isotopically labeled standards, and 2 mL of the sample was directly injected into an online solid-phase extraction column coupled to a mass spectrometer. Antibiotics from a wide range of classes were targeted in this study based on their frequency of detection in other studies worldwide, and their usage from records obtained from the Ministry of Health in Kuwait. These included: macrolides, clarithromycin, azithromycin, and erythromycin; sulfonamides, sulfamethoxazole and trimethoprim; nitroimidazoles, dimetridazole, metronidazole, metronidazole-OH, and ronidazole; fluoroquinolones, ciprofloxacin, and ofloxacin; the antibiotic, tetracycline; and β-lactams and cefalexin. Method detection limits and method quantification limits for all compounds are reported in Table 2.

### 2.3. Statistical Analyses

As only a one-time integrated sample was collected at each sampling location for each antibiotic, it was not possible to use a full model including all the tested parameters (location (2 levels), influent/effluent (2 levels), time (4 levels), and type of antibiotic (13 levels) on the measured concentration. First, a one-way ANOVA model was used to compare the effect of the type of antibiotics on the measured concentration. No significant effects were observed among the different types of antibiotics (F_12,169_ = 2.07, *p* = 0.03). An ANOVA 3 model was used to test the impact of location, influent/effluent, and time on antibiotic concentration without discriminating between antibiotics. Data were log transformed to fulfill assumptions of normality according to the Kolmogorov–Smirnov test and equality of variance following the Levene median test. All statistical analyses were performed using the SAS software.

## 3. Results and Discussion

**Figure 1:**
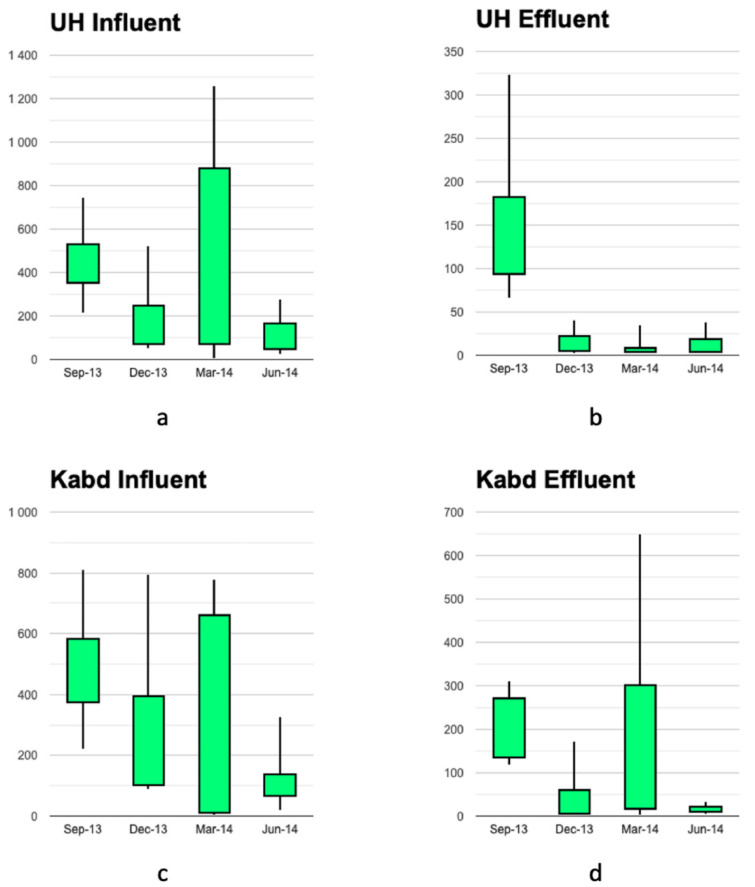
The concentration (µg/L) of various classes of antibiotics in the aqueous dissolved phase in the influents and effluents in two WWTPs in Kuwait.

The minimum, maximum, average, and median concentrations of individual antibiotics measured in dissolved aqueous influents and effluents of the two WWTPs studied are summarized in Table 3. Figure 1 presents average concentrations of various classes of antibiotics in the aqueous dissolved influents and effluents measured at both wastewater treatment plants. At the Umm Al Hayman WWTP, the dominant compounds in the aqueous dissolved influent were sulfamethoxazole with a mean concentration of 852 ng/L, ciprofloxacin (mean, 672 ng/L), clarithromycin (mean, 592 ng/L), and cefalexin (mean, 491 ng/L). At the Kabd WWTP, clarithromycin was the dominant compound in the influent at a mean concentration of 949 ng/L, followed in decreasing importance by ciprofloxacin (mean, 865 ng/L), cefalexin (mean, 598 ng/L), and sulfamethoxazole (mean, 520 ng/L). The dominant compounds in the dissolved effluent from Umm Al Hayman were sulfamethoxazole (mean, 212 ng/L), ciprofloxacin (mean, 153 ng/L), ofloxacin (mean, 120 ng/L), dimetridazole (mean, 96 ng/L), and metronidazole (mean, 93 ng/L). Whereas at the Kabd WWTP, the dominant compounds were sulfamethoxazole (mean, 338 ng/L), dimetridazole (mean, 274 ng/L), cefalexin (mean, 213 ng/L), ciprofloxacin (mean, 192 ng/L), and clarithromycin (189 ng/L). The concentrations measured in both aqueous dissolved influents and effluents in this study were higher than those reported by Gros et al. [39], for the same antibiotics in the WWTP influents and effluents from Girona, Spain. They were also higher than the median concentrations reported in the database compiled for the 117 WWTPs worldwide. These concentrations were also comparable to the effluent concentrations reported from 13 WWTPs in Portugal (PT), Spain (ES), Cyprus (CYP), Ireland (IL), Germany (DE), Finland (FI), and Norway (NO) [9].

The mean influent concentrations of all compounds were higher than those measured in the effluents. The concentration of fluoroquinolone antibiotics was the highest in the influent followed in decreasing order of importance by sulfonamides, macrolides, nitroimidazoles, β-lactams, and tetracycline. In the effluents, however, the concentrations of nitroimidazoles were dominant, particularly at Kabd, followed in decreasing order of importance by sulfonamides, fluoroquinolones, β-lactams, macrolides, and tetracycline. The difference in concentrations of antibiotics between the two WWTPs was not statistically significant (*p* > 0.05). The mean concentrations were very similar for all the compounds except for cefalexin, ciprofloxacin, and clarithromycin. Higher mean concentrations of clarithromycin were measured at the Kabd WWTP and of sulfamethoxazole at the Umm Al Hayman WWTP.

The differences in the influent concentrations observed between two WWTPs may be influenced by government policies on housing and drug prescription implemented in Kuwait. Foreign workers primarily inhabit certain areas, and certain medicines are only prescribed to Kuwaiti nationals. The differences observed in the influent concentrations between the two WWTPs may be attributed to the catchment of the wastewater reaching the plant. The effluent concentrations of all compounds were higher in the aqueous phase at Kabd as compared with the concentrations measured at the Umm Hayman WWTP, except for metronidazole, metronidazole-OH, ofloxacin, and ranidazole where the concentrations were identical for both WWTPs. This observation appears to suggest that the Umm Hayman WWTP may be a more efficient plant at attenuating the concentrations of antibiotics as compared with the Kabd WWTP.

An ANOVA 3 model revealed a significant effect of the influent/effluent and time but no effect of the location or any interaction (Table 4). The concentration of antibiotics was significantly higher in influent waters as compared with effluent waters. Scheffe’s post hoc test revealed that the concentration of antibiotics was significantly higher in September 2013 as compared with the three other sampling times.

### 3.1. Removal Efficiencies from the Influents of the Two WWTPs

The removal efficiency, which was computed as the percent reduction between the dissolved aqueous phase concentration of each compound in the influent and the dissolved aqueous phase concentration of the same compound in the effluent (Equation (1)) is presented in Table 5.

(1) $$Removal\;Effeciency=\frac{Influent\;Concentration\;–Effluent\;Concentration}{Influent\;Concentration}\times 100$$

The removal of antibiotics from the influents of WWTPs has been reported to vary widely worldwide. It depends heavily on the type of compound, the operating conditions, and the treatment technology [37,68,69,70]. The removal of antibiotics in WWTPs can occur by volatilization, sorption on sludge or particulate matter (by hydrophobic or electrostatic interaction), and degradation primarily by bacterial breakdown (e.g., oxidation, hydrolysis, demethylation, and cleavage of conjugates). It is, however, thought that volatilization of most antibiotics is a negligible removal pathway in WWTPs [71,72,73]. The most critical parameter controlling the bacterial removal of antibiotics and other pharmaceuticals is the retention time of the sludge in the WWTP [74]. The concentrations of antibiotics over four sampling campaigns at the two WWTPs are given in Table 6. The removal efficiencies of the various antibiotics over the four sampling campaigns for the Kabd and Umm Hayman WWTPs showed that they were variable and compound dependent, with removal efficiencies ranging between 10.87 and 99.75%. There are also subtle differences between the treatment plants. The Umm Hayman WWTP had higher removal efficiencies for most compounds than the Kabd WWTP. The reason for the higher performance of the Umm Hayman WWTP was a higher retention time; the Kabd WWTP is a more recent and advanced plant that serves a very big catchment area, and the sludge retention time is comparatively shorter than Umm Hayman. The most efficiently removed compounds were azithromycin > ciprofloxacin > trimethoprim > ofloxacin, all with removal efficiencies above 60% in both the WWTPs. In contrast, those with the lowest removal efficiencies were erythromycin, ronidazole, and tetracycline. The oxidation removal with UV shows a removal of 95% antibiotics [75], in the case of Kuwait where the summer temperatures and UV radiation are too high.

### 3.2. Temporal Variability in the Concentration of Antibiotics

Several studies have reported seasonal variations in the concentrations of different medications in WWTP influents and effluents, and surface water [73]. These changes have been linked to differences in consumption patterns in the case of influent concentrations, since certain drugs are consumed more at specific times of the year to cure certain seasonal ailments [55,68,73,76,77,78,79,80]. A typical example is the increased consumption of flu-related medications in winter in European countries. It has been argued that the effluent concentrations may be lower during summertime due to the enhanced microbial degradation during wastewater treatment in summer as compared with slower degradation in the colder winter months [55,68,76]. Some have argued that seasonality may be linked to increased rainfall with the potential to dilute effluent concentrations or reduce microbial activity in WWTP in Kuwait during each sampling campaign.

The concentrations of antibiotics in the influents and effluents of the two WWTPs for all four sampling campaigns were examined to tease out patterns related to seasonal consumption linked to illnesses in Kuwait. There are two main seasons in Kuwait: the summer season, which generally begins at the end of March and ends sometime in October, and the winter season, which runs from November to March. There is generally an increase in flu occurrences in Kuwait during the summer-winter, winter-summer changeovers. During the four sampling campaigns, the mean temperatures were: September 2013, 40 °C; December 2013, 13 °C; March 2014, 22 °C; and June 2014, 41 °C [81]. The March and September sampling periods may reflect these winter-summer and summer-winter transitions, respectively. The data clearly showed that the concentrations of antibiotics measured in the influents of both WWTPs were highest in samples collected during the winter-summer (September samples) transition followed by the concentrations measured during the summer-winter (March samples) transition period. This was possibly linked to the increased prescription of these medications to treat infectious diseases and flu prevalent in Kuwait during these/periods. The concentrations of various antibiotics were also dependent on the type of antibiotics, with erythromycin, ronidazole, tetracycline, azithromycin, and dimetridazole being more prevalent in the samples collected in September; clarithromycin, sulfamethoxazole, and ofloxacin mainly found in the samples collected in March.

## 4. Conclusions

This study was carried out to generate baseline concentrations of antibiotics in wastewater influents and effluents. The study also provided insight into the seasonality in the concentrations and a comparative assessment of the effectiveness of removing antibiotics during the treatment process at two WWTPs in regular operation in Kuwait. The concentrations of antibiotics measured in the influents of all WWTPs were considerably higher (almost an order of magnitude higher) than those measured in the effluents. The data also show that the concentrations of antibiotics in the dissolved influents and effluents in Kuwait are higher than the median concentrations of these compounds worldwide reported for 117 WWTPs but are comparable to levels reported in seven European countries. The higher level of antibiotics is possibly explained by the generous free healthcare available in-country and the advisory from the Ministry of Health in Kuwait to flush down unused/expired medication down the drain. For the first time, this study provides the concentrations of antibiotics in the dissolved aqueous influents and effluents of WWTPs in Kuwait. There is an eminent need to take up the comprehensive environmental impact that these residues may cause since there are instances when untreated wastewater is discharged directly into the marine environment. The discharge of treated or partly treated wastewater streams into the marine environment will possibly contribute to antibiotic resistance evolution. One of the highest concentrations observed in effluent was for ciprofloxacin that is notoriously known for its environmental risk and antibiotic resistance development. The treated effluent is also being utilized in agriculture farms and can lead to food chain transfer and the evolution of antibiotic-resistant microbes in the rhizosphere. There is an eminent need to undertake a detailed investigation of all the pharmaceuticals that are released into the marine environment.

## Figures and Tables

**Table 1:** Operational parameters of the wastewater treatment plants investigated.

	Umm Al Hayman WWTP	Kabd WWTP
Flow rate (m^3^/day)	27,000	180,000
Primary treatment	Screening and grit removal	Screening, grit removal, oil and grease removal
Secondary treatment	Aeration tanks (oxidation ditches)	Vertical activated sludge process
*Principle*	Extended aeration	Hybrid aerated anoxic-aerobic oxidation
*MLSS* (mg/L)	3000	6000
*SRT* (d)	25	8
*HRT* (h)	11	10
Secondary calcifiers		
*No tanks*	4	6
*MLSS* (mg/d)	2800	4000
*HRT* (h)	1.96	3.5
Tertiary treatment	Sand filtration	Disc filtration
*No. of units*	12	8
*Surface area* (m^2^)	20	100
Advance Treatment	Chlorination and UV radiation	Chlorination and UV radiation
*Residual chlorine* (mg/L)	0.5	0.5–1
*Population of catchment*	173,000	1,200,000

SRT, Solid retention time; HRT, hydraulic retention time; MLSS, mixed liquor suspended solids.

**Table 2:** Parameters indicating the performance of the analytical method: Method detection and quantification limits (MDL and MQL).

Compounds	Recoveries % (*n* = 3)		MDL (ng/L)		MQL (ng/L)	
	Effluent	Influent	WWE	WWI	WWE	WWI
Eryhromycin	137 ± 18.0	110 ± 18.0	1.1	2.1	3.5	6.9
Azithromycin	111 ± 11.6	74 ± 8.2	0.4	2	1.2	6.6
Clarithromycin	106 ± 12.5	75 ± 7.2	1.3	3.1	4.3	10.4
Tetracycline	127 ± 6.3	101 ± 2.2	7	26	23	60
Ofloxacin	116 ± 15.9	74 ± 14.3	0.6	3.7	1.8	12.1
Ciprofloxacin	140 ± 21.2	122 ± 21.9	7	10	23	35
Cefalexin	70 ± 8.6	108 ± 6.7	5	8	16.6	26.8
Sulfamethoxazole	81 ± 11.3	84 ± 5.5	5.5	7.1	18	23.7
Trimethoprim	67 ± 7.1	65 ± 6.9	2.4	7.1	8.1	20
Dimetridazole	109 ± 11.1	79 ± 2.0	15	20	50	68
Metronidazole	109 ± 4.7	127 ± 4.5	26	50	44	70
Metronidazole-OH	43 ± 8.2	61 ± 12.8	14	25	48	70
Ronidazole	108 ± 10.1	51 ± 2.8	15	17	51	53

WWE, wastewater effluents; WWI, wastewater influents.

**Table 3:** Summary of average, minimum, maximum, and median concentrations of antibiotics (*n* = 4), expressed in ng/L, in influents and effluents of the two wastewater treatment plants in Kuwait.

**Influent Concentration (ng/L)**								
	**Umm Al Hayman WWTP**				**Kabd WWTP**			
	**Average**	**Minimum**	**Maximum**	**Median**	**Average**	**Minimum**	**Maximum**	**Median**
Azithromycin	174	82	355	129	157	<MDL	466	78
Cefalexin	491	412	536	525	598	481	794	519
Ciprofloxacin	672	256	1335	548	865	237	1492	865
Clarithromycin	592	25	1258	493	949	37	1999	810
Dimetridazole	209	<MDL	415	210	169	<MDL	466	103
Erythromycin	111	6	216	111	112	<MDL	219	112
Metronidazole	238	145	331	238	246	144	356	236
Metronidazole-OH	128	30	365	59	184	96	384	128
Ofloxacin	446	64	889	415	446	87	779	460
Ronidazole	169	6	332	169	178	6	350	178
Sulfamethoxazole	852	264	1231	956	520	328	743	505
Tetracycline	293	48	537	293	249	21	562	164
Trimethoprim	262	117	419	257	234	112	479	172
**Effluent Concentration (ng/L)**								
	**Umm Al Hayman WWTP**				**Kabd WWTP**			
	**Average**	**Minimum**	**Maximum**	**Median**	**Average**	**Minimum**	**Maximum**	**Median**
Azithromycin	26	<MDL	74	14	48	6	119	32
Cefalexin	69	<MDL	203	2	213	<MDL	444	192
Ciprofloxacin	153	<MDL	533	40	192	<MDL	535	115
Clarithromycin	39	<MDL	80	35	189	14	420	134
Dimetridazole	96	<MDL	237	48	274	76	527	246
Erythromycin	34	<MDL	66	34	57	25	118	28
Metronidazole	93	29	157	93	93	34	182	64
Metronidazole-OH	62	<MDL	165	18	61	20	179	23
Ofloxacin	120	<MDL	324	76	127	<MDL	311	98
Ronidazole	80	<MDL	156	80	78	<MDL	153	78
Sulfamethoxazole	212	105	272	236	338	174	649	264
Tetracycline	75	24	126	75	112	<MDL	307	27
Trimethoprim	41	<MDL	141	10	48	6	131	28

**Table 4:** Summary of an ANOVA 3 (F and *p* values) testing the impact of location, influent/influent, and time on antibiotic concentration.

Source	F	*p*
Model	F_12,169_ = 10.05	<0.0001
Location	F_1_ = 1.98	0.16
Influent/effluent	F_1_ = 75.18	<0.0001
Time	F_1_ = 118.32	<0.0001
Location × influent/effluent	F_1_ = 1.86	0.17
Location × time	F_3_ = 0.16	0.93
Influent/effluent × time	F_3_ = 2.38	0.07
Location x influent/effluent × time	F_3_ = 0.76	0.52

**Table 5:** Removal efficiencies of antibiotics, expressed as percentages, during wastewater treatment over the four sampling campaigns at Kabd and Umm Hayman WWTPs.

	Umm Hayman				Kabd			
	September 2013	December 2013	March 2014	June 2014	Sepember 2013	December 2013	March 2014	June 2014
Azithromycin	79.08	85.40	89.93	97.79	74.41	66.60	87.38	86.45
Cefalexin	62.15	99.62	99.51		60.00	99.75	14.42	
Ciprofloxacin	60.10	83.97	99.51	94.47	64.13	13.14	99.70	97.70
Clarithromycin	83.87		97.23	91.86	83.50		78.98	62.97
Dimetridazole	42.93	91.67		99.05	44.75	85.67	98.29	98.02
Erythromycin	69.61		67.74		46.26		85.71	
Metronidazole	52.42	80.07			49.03	73.01		76.63
Metronidazole-OH	54.82	64.85		93.42	53.43	75.39	82.87	84.21
Ofloxacin	53.34	96.88	84.67	88.33	60.05	97.69	75.69	95.97
Ronidazole	53.00		50.79		56.39		45.16	
Sulfamethoxazolle	85.98	10.87	77.95	79.64	52.52	56.36	12.59	27.44
Tetracycline	76.55			50.06	45.40	98.78		21.79
Trimethoprim	66.28	84.89	99.15	99.28	72.75	81.41	85.50	94.49

**Table 6:** Concentrations of antibiotics, expressed in ng/L, in influents and effluents of the two wastewater treatment plants in Kuwait, sampled at four different seasons of the year.

	Umm Al Hayman WWTP								Kabd WWTP							
	Influent				Effluent				Influent				Effluent			
	September 2013	December 2013	March 2014	June 2014	September 2013	December 2013	March 2014	June 2014	September 2013	December 2013	March 2014	June 2014	September 2013	December 2013	March 2014	June 2014
Azithromycin	355.2	81.5	166.8	90.66	74.3	11.9	16.8	2	465.8	94	31.7	62.2	119.2	31.4	4	8.43
Cefalexin	535.5	524.6	412.3	Nd	202.7	2	2	nd	480.5	794.2	518.9	nd	192.2	2	444.1	nd
Ciprofloxacin	1335.4	255.8	406.7	688.34	532.8	41	2	38.08	1491.8	236.7	665.6	1064.9	535.1	205.6	2	24.53
Clarithromycin	492.9	Nd	1258.1	24.57	79.5	nd	34.8	2	810.1	Nd	1998.9	37.29	133.7	nd	420.1	13.81
Dimetridazole	414.6	48	Blq	209.62	236.6	4	blq	2	466.4	527	233.97	202.2	257.7	75.5	4	4
Erythromicin	215.5	Nd	6.2	Nd	65.5	nd	2	nd	219.4	Nd	28	nd	117.9	nd	4	25.4
Metronidazole	330.6	145	Blq	Nd	157.3	28.9	blq	nd	356.3	236	blq	144.28	181.6	63.7	blq	33.72
Metronidazole-OH	365	51.5	65.5	30.41	164.9	18.1	nd	2	384.4	96.3	130.2	126.69	179	23.7	22.3	20.01
Ofloxacin	695.2	64.1	888.5	135.34	324.4	2	136.2	15.79	777.8	86.7	779.1	141.38	310.7	2	189.4	5.7
Ronidazole	331.9	Nd	6.3	Nd	156	nd	3.1	nd	350.4	Nd	6.2	nd	152.8	nd	3.4	Nd
Sulfamethoxazolle	747.6	264.1	1231.1	1163.94	104.8	235.4	271.5	236.92	612	398.3	742.9	328.13	290.6	173.8	649.4	238.1
Tetracycline	536.9	Nd	Blq	48.16	125.9	nd	blq	24.05	561.5	164.4	blq	26.8	306.6	2	blq	20.96
Trimethoprim	419.3	116.5	235.9	278.14	141.4	17.6	2	2	479.2	171.6	172.4	111.88	130.6	31.9	25	6.17

## Data Availability

The complete data is provided in the paper.

## Supplementary Materials

The following supporting information can be downloaded at: https://www.mdpi.com/article/10.3390/toxics10040174/s1, Table S1: ES+Tuning parameters for the target analytes on the AQUITY UPLC—Xevo TQS System title, Table S2: ES-Tuning parameters for the target analytes on the AQUITY UPLC—Xevo TQS System.
