# Supplementary material for: Antibiotics in Wastewater: Baseline of the Influent and Effluent Streams in Kuwait
Source: Toxics. 2022 Apr 1;10(4):174. doi: 10.3390/toxics10040174 (PMC9025492; doi:10.3390/toxics10040174)
Supplement: Supplementary file 1 [file toxics-10-00174-s001.zip › toxics-1613478-supplementary.pdf]

toxics

Article

Antibiotics in Wastewater: Baseline of the Influent and Effluent Streams in Kuwait

Bondi Gevao [1], Saif Uddin [2,*], Divya Krishnan [2], Smitha Rajagopalan [2] and Nazima Habibi [2]

[1] Environmental Protection Authority, Freetown 47235, Sierra Leone; bondi.gevao@epa.gov.sl
[2] Environment and Life Sciences Research Center, Kuwait Institute for Scientific Research, Safat 13109, Kuwait; dkrishnan@kisr.edu.kw (D.K.); srajagopalan@kisr.edu.kw (S.R.); nhabibi@kisr.edu.kw (N.H.)
* Correspondence: sdin@kisr.edu.kw

Method Development

During the period covered by this report a new UPLC-MS/MS system (AQUITY UPLC- Xevo TQ-S) has been installed providing improved analytical capabilities due to a 10 times lower instrumental detection limit. The initial draft method has been reviewed and optimized to take full advantage of the new instrumental capabilities. Mixtures of the target compound have been infused and the tune parameters optimized. In tables 1 and 2 we report the experimentally optimized tuning parameters for each of the target compounds (and the respective labeled analogues) under the following common instrumental conditions:

Capillary Voltage (kV)	0.2
Source offset (V)	60
Desolvation Temperature (°C)	400
Desolvation (L/Hr)	800
Cone(L/Hr)	150
Nebuliser(Bar)	7

Citation: Gevao, B.; Uddin, S.; Krishnan, D.; Rajagopalan, S.; Habibi, N. Antibiotics in Wastewater: Baseline of the Influent and Effluent Streams in Kuwait. *Toxics* **2022**, *10*, 174. https://doi.org/10.3390/toxics10040174

Academic Editor: Roberto Rosal

Received: 10 February 2022
Accepted: 15 March 2022
Published: 1 April 2022

Publisher's Note: MDPI stays neutral with regard to jurisdictional claims in published maps and institutional affiliations.

Copyright: © 2022 by the authors. Licensee MDPI, Basel, Switzerland. This article is an open access article distributed under the terms and conditions of the Creative Commons Attribution (CC BY) license (https://creativecommons.org/licenses/by/4.0/).

Table S1. ES+Tuning parameters for the target analytes on the AQUITY UPLC – Xevo TQS System.

Compound	Detection mode	Center Mass Parent	Cone Voltage	Center mass quantifier	Collision energy	Center mass qualifier	Collision energy
Acetaminophen	ES+	152	30	93	20	110	18
Acetaminophen -$^{13}C_2$	ES+	155	30	93	19	111	16
Caffeine	ES+	194.95	20	110	25	138.15	18
Caffeine-$^{13}C_3$	ES+	198	32	98	25	140.05	19
Carbamazepine	ES+	237	20	179	35	194.05	20
Carbamazepine- d_{10}	ES+	247.05	20	159	18	204.2	35
Erythromycin	ES+	734.6	30	158.15	32	576.5	18
Erythromycin-$^{13}C_2$	ES+	736.5	30	160.1	30	578.4	19
Testosterone	ES+	289.1	30	143	18	201.2	17
Testosterone-d_5	ES+	293.1	30	199.1	30	228.15	30
Sulfamethoxazole	ES+	254	5	92.2	35	155.95	30
Sulfamethoxazole-$^{13}C_6$	ES+	259.1	15	171.1	16	241	8
Fluoxetine HCl	ES+	310.1	20	44	8	148.05	7
Fluoxetine HCl -d_6	ES+	316.25	5	44.05	10	154.1	8
Ciprofloxacin-HCl	ES+	332.2	40	119.05	25	231.1	15
Ciprofloxacin-HCl -$^{13}C_3$	ES+	336.25	20	235.05	35	304.1	20
Progesterone	ES+	315.2	30	97	20	109.05	20
Progesterone-d_9	ES+	323.05	25	100.2	20	113.2	30

Table S2. ES-Tuning parameters for the target analytes on the AQUITY UPLC – Xevo TQS System.

Compound	Detection mode	Center Mass Parent	Cone Voltage	Center mass quantifier	Collision energy	Center mass qualifier	Collision energy
Triclosan	ES-	286.9	20	35	5	37	5
Triclosan $^{13}C_{12}$	ES-	298.9	20	35	5	37	5
Ibuprofen	ES-	204.85	20	161	10		
Ibuprofen-$^{13}C_3$	ES-	209	30	164.15	8		
Estrone	ES-	269.05	60	145.1	35	183.05	35
Estrone-$^{13}C_2$	ES-	271.1	30	148.1	40	185.05	35
Estradiol	ES-	271.1	20	145	40		
Estradiol-$^{13}C_2$	ES-	272.9	30	146.95	40		
Ethinyl Estradiol	ES-	295.1	20	145.05	38		
Ethinyl Estradiol-$^{13}C_2$	ES-	297.1	50	145.05	38		
Diclofenac	ES-	293.9	20	214	20	250	12
Diclofenac- d_4	ES-	298.91	30	255	12		
Gemfibrozil	ES-	249	25	121	15	127	10
Gemfibrozil-d_6	ES-	255	30	121.02	20		
Naproxen	ES-	229	30	170	15	185.1	10
Naproxen- ^{13}C	ES-	233.05	30	169.05	30		

Subsequently a set of runs with different gradient settings for both ES+ and ES- detection mode were carried out to optimize the chromatographic separation, peak shape and simplify detection in the chromatographic areas in which was expected potential superposition.

Following are reported the optimized gradient and inlet parameters for each detection mode

ES-

Mobile Phases:

A- 5mm ammonium acetate in ultra pure water
B- 10/90 v/v water /acetonitrile with 5mm ammonium acetate

Gradient:

Step	Time (min)	Flow(ml/min)	%A	%B	%C	%D	Curve
1	0.0	0.4	80	20	0	0	
2	4.5	0.4	4	96	0	0	6
3	5	0.4	0	100	0	0	6
4	6.21	0.4	80	20	0	0	6
5	7.8	0.4	80	20	0	0	6

Total run time = 8 minutes.

Seal wash = 1 minute.

ES+

Mobile Phases:

A- Ultra pure water with 0.1% formic acid
B- Acetonitrile with 0.1% formic acid

Gradient:

Step	Time (min)	Flow(ml/min)	%A	%B	%C	%D	Curve
1	0.0	0.4	95	5	0	0	
2	1.5	0.4	95	5	0	0	6
3	3	0.4	80	20	0	0	6
4	4	0.4	55	45	0	0	6
5	6.1	0.4	35	65	0	0	6
6	7	0.4	0	100	0	0	6
7	7.2	0.4	0	100	0	0	6
8	7.45	0.4	95	5	0	0	6
9	8.9	0.4	95	5	0	0	6

Total run time = 9 minutes.

Seal wash = 1 minute.

The linear dynamic range of the instrument response was then verified by injecting a set of standards containing the target analytes, and their labeled analogues, at the following increasing concentrations: 10, 20, 40, 50 ,60 ,80, 100 and 200 ppb (concentration of each component in the mixture).Saturation trend has been observed for some of the target analytes at concentration of 200 ppb resulting in a loss of response linearity. It has been noticed that all the analytes exhibit suitable response linearity ($R^2 > 0.995$) in the calibration range 10 to 100 ppb. In figure 1 is reported one of the target analyte calibration curve with maximum calibration standard at 100 ppb and related correlation coefficient.
